# Kidney Organoids and Tubuloids

**DOI:** 10.3390/cells9061326

**Published:** 2020-05-26

**Authors:** Fjodor A. Yousef Yengej, Jitske Jansen, Maarten B. Rookmaaker, Marianne C. Verhaar, Hans Clevers

**Affiliations:** 1 Hubrecht Institute—Royal Netherlands Academy of Arts and Sciences and University Medical Center Utrecht, Uppsalalaan 8, 3584 CT Utrecht, The Netherlands; f.yousef@hubrecht.eu; 2 Department of Nephrology and Hypertension, University Medical Center Utrecht, Heidelberglaan 100, 3584 CX Utrecht, The Netherlands; m.rookmaaker@umcutrecht.nl (M.B.R.); m.c.verhaar@umcutrecht.nl (M.C.V.); 3 Department of Pathology, Radboud Institute for Molecular Life Sciences, Radboud University Medical Center, Geert Grooteplein 24, 6500 HB Nijmegen, The Netherlands; jitske.jansen@radboudumc.nl; 4 Department of Pediatric Nephrology, Radboud Institute for Molecular Life Sciences, Radboud University Medical Center, Amalia Children’s Hospital, Geert Grooteplein 24, 6500 HB Nijmegen, The Netherlands

**Keywords:** kidney, organoids, tubuloids, pluripotent stem cells, adult stem cells

## Abstract

In the past five years, pluripotent stem cell (PSC)-derived kidney organoids and adult stem or progenitor cell (ASC)-based kidney tubuloids have emerged as advanced in vitro models of kidney development, physiology, and disease. PSC-derived organoids mimic nephrogenesis. After differentiation towards the kidney precursor tissues ureteric bud and metanephric mesenchyme, their reciprocal interaction causes self-organization and patterning in vitro to generate nephron structures that resemble the fetal kidney. ASC tubuloids on the other hand recapitulate renewal and repair in the adult kidney tubule and give rise to long-term expandable and genetically stable cultures that consist of adult proximal tubule, loop of Henle, distal tubule, and collecting duct epithelium. Both organoid types hold great potential for: (1) studies of kidney physiology, (2) disease modeling, (3) high-throughput screening for drug efficacy and toxicity, and (4) regenerative medicine. Currently, organoids and tubuloids are successfully used to model hereditary, infectious, toxic, metabolic, and malignant kidney diseases and to screen for effective therapies. Furthermore, a tumor tubuloid biobank was established, which allows studies of pathogenic mutations and novel drug targets in a large group of patients. In this review, we discuss the nature of kidney organoids and tubuloids and their current and future applications in science and medicine.

## 1. Introduction

Kidney disease affects around 15% of the population and is accompanied by a substantial increase in morbidity and mortality [1]. New therapeutic approaches are urgently needed, but their development is hampered by the high complexity of the kidney. Human primary and immortalized cell lines have enabled detailed investigation of specific cell types, but lack histological characteristics of the kidney. Furthermore, immortalization interferes with the assessment of the cell cycle and related diseases. On the other hand, animal experiments provide a more physiological model, but are troubled by ethical issues and translational constraints due to interspecies differences. To further advance our understanding of renal pathophysiology and facilitate the development of novel and personalized therapies, advanced in vitro models that use patient-derived renal tissue and better mimic the nephron are required.

In the past decade, organoid cultures have rapidly emerged as a powerful in vitro model for many different organs. Organoids are three-dimensional (3D) multicellular cultures that resemble the structure, physiology and diseases of their organ of origin. These cultures are generated from either pluripotent stem cells (PSC) or adult stem or progenitor cells (ASC), two complementary techniques with their own characteristics and specific fields of application. Organoids have been established from organs within the digestive tract, airways, urogenital tract, reproductive system, and central nervous system. Both organoid types have been used to study organ physiology and disease and to test new interventions including drugs and gene therapy. Drug efficacy screenings have been performed in biobanks containing organoids from large groups of patients to find a general treatment for a heterogenic patient population, and in organoids from specific patients to provide personalized medicine. Furthermore, the possibility to use organoid-based cells and tissues in regenerative medicine is being explored [2,3].

In the past five years, the processes of nephrogenesis and homeostasis and regeneration in the adult kidney have been recapitulated in the laboratory to generate PSC and ASC-derived kidney organoids [4,5,6,7,8,9]. These organoids hold great potential to improve our understanding of kidney disease and facilitate the discovery of new therapeutic options. In this review, we will first briefly summarize the complex architecture and physiology of the kidney to stress the key advantages of organoids in nephrology. Next, we will discuss renal development, homeostasis, and regeneration, as these processes guide organoid culture. Finally, we will focus on the nature of PSC and ASC-derived kidney organoids and their current and future applications in science and medicine.

## 2. Kidney Physiology, Development and Regeneration

### 2.1. Renal Anatomy and Physiology

**Figure 1:**
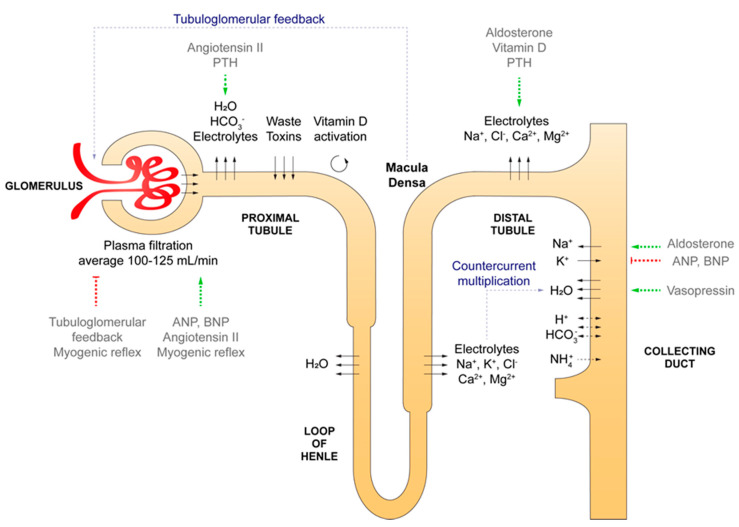
Summary of the main filtration, reabsorption, excretion and endocrine processes across the nephron. Important hormones and mechanisms that regulate these functions are depicted in grey. ANP = atrial natriuretic peptide. BNP = brain natriuretic peptide. PTH = parathyroid hormone.

A human kidney consists of intricate functional units named nephrons that originate in the renal cortex and stretch out into the renal medulla. Nephrons are responsible for most functions of the kidney, including (1) regulation of electrolyte, acid-base, and volume homeostasis, (2) filtration and excretion of toxins, metabolic waste products, and xenobiotics, whilst reabsorbing nutrients, water and electrolytes from the filtrate, (3) endocrine activity, and (4) interaction with the sympathetic nervous system. The nephron consists of over 20 different cell types that cluster in five major segments, each with their specialized functions (Figure 1). The first segment is the glomerulus, a size and electric charge-specific barrier that filtrates plasma to create pro-urine. The glomerular filtration barrier is formed by fenestrated endothelial cells, a basement membrane and the foot processes of podocytes. The parietal epithelial cells in Bowman’s capsule are continuous with the epithelium of the proximal tubule. The proximal tubule has a leaky epithelium and employs a wide array of specialized transporters to reabsorb the bulk of the filtered water, electrolytes, bicarbonate, and nutrients. This segment also excretes toxins and xenobiotics. In addition, vitamin D is activated in the proximal tubule. The next segment is the loop of Henle, which descends into the renal medulla and then ascends back into the cortex. The descending limb mostly reabsorbs water, whereas the ascending limb and the following distal tubule are mainly involved in electrolyte reabsorption. The final part of the nephron is the collecting duct, which consists of principal cells and intercalated cells. Principal cells reabsorb water and sodium (in exchange for potassium) under regulation of vasopressin and aldosterone, respectively. Intercalated cells subdivide in α- and β-intercalated cells, which specialize in respectively proton and bicarbonate excretion to maintain acid-base homeostasis. Although nephron epithelium is responsible for the majority of functions, stromal cells also have important roles. For example, pericytes support peritubular capillaries and mesangial cells organize glomerular capillary loops and remove debris. Furthermore, smooth muscle cells regulate vascular tone in the arterioles. These cells are required to sustain the renal vasculature, which is indispensable for filtration, excretion, and reabsorption. The complex architecture of the renal epithelium and vasculature are reinforced by the fibroblast-rich renal interstitium, which also hosts immune cells. Two particularly intriguing stromal populations are specialized peritubular fibroblasts that produce the hormone erythropoietin and juxtaglomerular cells around the afferent arteriole that secrete renin.

### 2.2. Nephrogenesis

**Figure 2:**
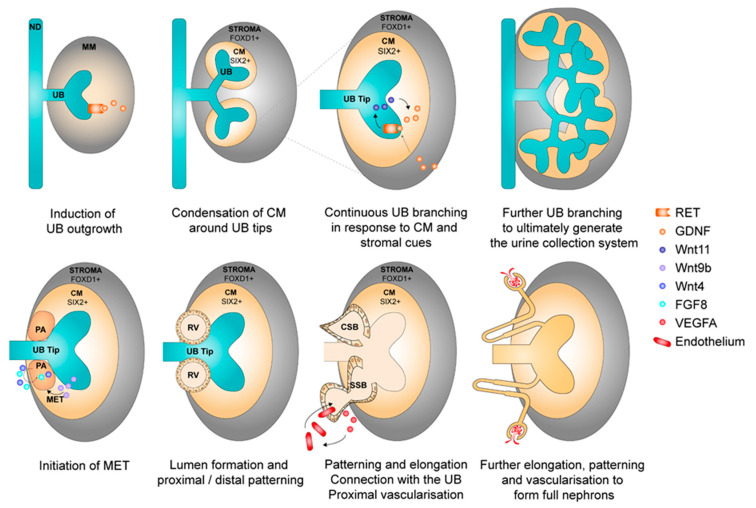
Brief overview of kidney development and some of the key signals that regulate specific steps. The top figures display induction and maintenance of ureteric bud branching. The bottom figures display the transition of mesenchyme to epithelial structures and subsequent elongation, patterning and vascularization to generate the full nephron. ND = nephric duct. UB = ureteric bud. MM = metanephric mesenchyme. CM = cap mesenchyme. PA = pretubular aggregate. MET = mesenchymal-to-epithelial transition. RV = renal vesicle. CSB = comma-shaped body. SSB = S-shaped body. GDNF = glial cell line-derived neurotrophic factor. FGF8 = fibroblast growth factor 8. VEGFA = vascular endothelial growth factor A.

Nephron formation takes place during gestation and results in the generation of on average 1 million nephrons per kidney, with large variations between individuals. The kidney is derived from the mesoderm germ layer, more specifically from the anterior mesoderm-derived ureteric bud (UB) and posterior mesoderm-derived metanephric mesenchyme (MM) precursor tissues. The UB and MM exchange reciprocal signals to induce, maintain, and complete nephrogenesis (Figure 2). This process starts with budding outgrowth from the nephric duct (or Wolffian duct) under the influence of signals (mainly glial cell line-derived neurotrophic factor, GDNF) from the surrounding MM. The budding forms the UB, which starts to sprout into the MM. The surrounding MM condenses around the sprouting UB tips to form the SIX2+ cap mesenchyme (CM). A positive feedback loop between CM secreted GDNF and Wnt11 from the UB tip ensures further dichotomous sprouting of the UB to eventually form the collecting ducts and ureters. In the meantime, the UB also secretes Wnt9b, which causes CM cells to differentiate to the pretubular aggregate (PA). In the PA, fibroblast growth factor (FGF) 8 and Wnt4 signals induce LIM homeobox 1, which in turn initiates mesenchymal-to-epithelial transition. This results in formation of a lumen: the renal vesicle (RV) stage. The RV subsequently starts to bend, elongate, and pattern to generate the comma-shaped body and subsequently the S-shaped body. Various signals are involved in the patterning process, including Wnt4, FGF and Notch signaling. In the S-shaped body, production of vascular endothelial growth factor isoforms attracts endothelial cells to the proximal part to ultimately form the glomerulus. Endothelial cells in turn produce platelet-derived growth factor subunit B to attract mesangial progenitor cells that organize endothelial cells in glomerular capillary loops. The S-shaped body continues to differentiate and transforms into the mature nephron [10,11,12]. The stromal part of the kidney derives from forkhead box D1 (FOXD1)+ stromal progenitors that emerge in the periphery of the metanephric mesenchyme and to a lesser extent invading TBX18+ progenitors. These FOXD1+ and TBX18+ cells give rise to fibroblasts, smooth muscle, pericytes, and mesangial cells. Furthermore, during kidney development, the stromal population provides cues (e.g., GDNF) that modulate UB branching and cap mesenchyme differentiation [13,14,15,16,17,18,19]. Nephron formation permanently ceases around week 36 of gestation.

### 2.3. Injury and Regeneration in the Adult Kidney

**Figure 3:**
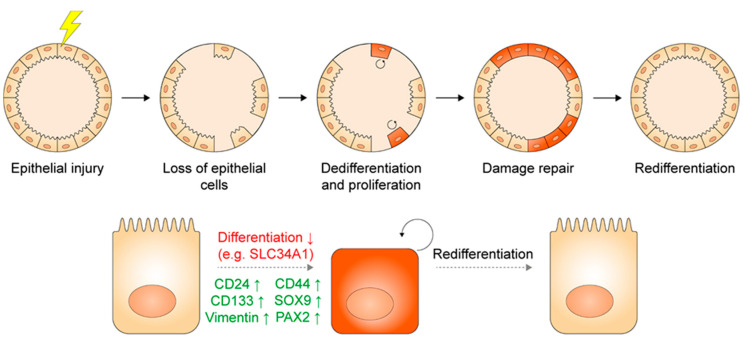
Proposed mechanism for regeneration in the adult proximal tubule. Upon injury, generic differentiated epithelial cells dedifferentiate, proliferate and redifferentiate to repair the damage. During this process, differentiated cells lose markers of differentiation (e.g., the brush border, specific transporters) and upregulate markers that indicate a stem or progenitor cell state (CD24, CD133, Vimentin and probably CD44, SOX9 and PAX2 as well).

During homeostasis, the kidney shows a low rate of proliferation. However, upon injury, the tubular part of the nephron has significant capacity to repair itself by a burst of proliferation [20]. Various studies suggest that this process is mediated by proliferating tubular epithelial cells that are characterized by CD24, CD133, Vimentin, SOX9, CD44, and possibly PAX2 expression [21,22,23,24,25,26,27,28,29,30]. These proteins could mark either a constitutively present population of progenitor cells that activate during renewal and repair, or mark a stem cell state that differentiated cells could adapt, as is the case for several other organs [31,32]. This remains a matter of debate in the field [20,25,33]. An elegant lineage tracing study provides insight in this matter. Kusaba and colleagues found that labeled SLC34A1+ differentiated proximal tubule cells start to proliferate and repair that segment after ischemia reperfusion injury. During this process, labeled cells downregulated SLC34A1 and upregulated CD24, CD133, and Vimentin. These data indicate that a plastic process of dedifferentiation, proliferation, and redifferentiation is responsible for renal repair, at least in the proximal tubule [24,25]. This is in line with other studies that also advocate a process of plasticity rather than a constitutively present progenitor population [21,22,34,35,36]. Based on these findings, CD24, CD133, Vimentin, and probably the other aforementioned markers (CD44, SOX9, PAX2) appear to mark a progenitor state that differentiated cells can adopt upon damage (Figure 3). Another question is whether tubular repair is mediated by segment-committed or more promiscuous progenitors. Rinkevich and colleagues used inducible Confetti labeling to trace the fate of tubular epithelial clones during nephrogenesis and both homeostasis and repair in the adult kidney. Labeled clones predominantly expanded in a proximal tubule, loop of Henle, distal tubule, or collecting duct-committed manner. Interestingly, Wnt signaling appears important in tubular regeneration, since clones expressing the Wnt target gene Axin2 established larger clonal populations upon damage than unselected labeled cells. Wnt-responsive cells contributed only to proximal tubules and collecting ducts [37]. Other studies support an important role for Wnt in the adult kidney as well [38]. Segment-committed Wnt-responsive progenitors also play a role during nephrogenesis, where LGR5+ cells arise during the S-shaped body stage and contribute to formation of the loop of Henle and distal tubule [39]. Besides Wnt, other signaling pathways contribute to tubular epithelial regeneration as well, especially tyrosine kinase signals including epidermal growth factor (EGF), FGFs, and insulin-like growth factor 1. These signals require precise timing and dosing as overexposure can result in maladaptive repair with subsequent fibrosis [40,41].

Acute kidney injury (AKI) can result from numerous causes, including nephrotoxic agents, ischemia, infections, immune-mediated tubulo-interstitial nephritis and auto-immune disease. Although the kidney has a high regenerative ability, damage can surpass this capacity and become irreversible, resulting in permanent loss of nephrons. Nonetheless, the accompanying decline in kidney function is often ameliorated by compensatory hypertrophy of the remaining nephrons. Repeated AKI episodes and more insidious chronic damage (e.g., hypertension or diabetic nephropathy) can exceed the compensatory potential of the kidney and/or elicit maladaptive repair, both leading to chronic kidney disease (CKD). This is a common endpoint characterized by epithelial senescence, pericyte detachment, accumulation of myofibroblasts, loss of microvasculature, and progressive fibrosis. One of the suspected driving factors are senescent epithelial cells that adopt a profibrotic and proinflammatory secretory phenotype. Senescent cells arise from proliferating cells that are ageing or that face DNA damage and subsequent cell cycle arrest without adequate repair or initiation of apoptosis. The origin of pathogenic myofibroblasts is debated, and suspected sources include pericytes and interstitial fibroblasts. Episodes of AKI increase the risk of CKD, but also vice versa [13,42,43,44,45,46,47,48,49].

## 3. Kidney Organoids and Tubuloids

The intricate architecture and the many cell types and complex interactions involved in kidney development, repair and function require advanced tools for in-depth studies. Kidney organoids and tubuloids are established from respectively PSC and ASC. These are 3D multicellular structures that more accurately resemble the complex architecture and composition of the kidney in vivo than cell lines. Both organoid types also better reflect human physiology and disease compared to animal models and allow personalized medicine and high-throughput drug screening. In the following paragraphs, kidney organoids and tubuloids will be discussed in detail.

### 3.1. Pluripotent Stem Cell-Derived Kidney Organoids

#### 3.1.1. Sources of Pluripotent Stem Cells

Pluripotent stem cell-derived organoids are established from either embryonic stem cells (ESC) or induced PSC (iPSC). Embryonic stem cells were first isolated and cultured in vitro from mouse embryos in 1981 and from human blastocysts in 1998 [50,51,52]. These are self-renewing cells that have the potential to differentiate into cell types of all germ layers (endoderm, mesoderm and ectoderm) [52]. In 2006 and 2007, iPSC that harbor the same potency for self-renewal and differentiation as ESC were generated by dedifferentiating mouse and human fibroblasts via retroviral transfection with four transcription factors: Sox2, Oct3/4, Klf4, and c-Myc [53,54]. The development of iPSC took away the ethical concerns of ESC and provided an autologous cell source. Since then, innovative novel reprogramming techniques have been developed and used, including non-genome-integrating viruses, plasmids, micro RNAs, synthetic messenger RNAs, and the injection of recombinant proteins [55,56,57,58,59,60,61,62,63]. Many cell types can be reprogrammed to iPSC, including relatively easily accessible skin fibroblasts, adipocytes, peripheral blood cells and epithelial cells from urine [63,64]. The latter is advantageous because the procedure is non-invasive. Furthermore, since the epigenetic memory of donor cells influences iPSC differentiation, the use of (urine-derived) renal cells appears favorable for the creation of kidney organoids [65,66].

#### 3.1.2. Generation and Characterization of Pluripotent Stem Cell-Derived Kidney Organoids

In vivo studies have greatly expanded our knowledge about nephrogenesis and are the foundation of differentiation protocols that allow generation of kidney organoids from iPSC. Based on these studies, it is known that the reciprocal interaction between UB and MM that is governed by growth factors secreted by these populations (e.g., Wnt11, Wnt9b, FGF) is essential for nephrogenesis. These signals are mimicked in vitro to push iPSC towards primitive streak and intermediate mesoderm and subsequently towards UB, MM or both. The UB and MM populations then interact with one another, resulting in further differentiation and self-organization into nephron structures [4,5,6,7,67,68,69,70,71,72,73,74,75,76,77,78]. To date, various well characterized protocols to generate kidney organoids are available. The predominant factor used in these protocols is the activation of canonical Wnt signaling, mostly via CHIR99021 [4,5,6,7,11]. The duration of Wnt signaling determines whether UB or MM becomes the most dominant population, though often an intermediate condition is used to obtain both populations. Consecutive addition of FGF9 then enables MM patterning. Together, this results in the formation of complex multicellular 3D renal organoids that consist of approximately 500 nephron-like structures [74]. Further protocol refinement allowed more selective differentiation into UB and MM alone. Subsequent combination of separately cultured UB and MM populations with embryonal stromal cells resulted in higher-order organized mouse renal organoids with an interconnected urine collecting system. The stromal population appears to provide one of the distinct cues essential to generate high-order organogenesis in rodent organoids [76].

Organoids have been extensively characterized. Single cell RNA-sequencing revealed that organoids contain developing podocytes, parietal epithelial cells, proximal tubules, loops of Henle, distal tubules, collecting ducts, and interstitial, endothelial, and stromal cells. Underrepresented or missing cell types include mesangial cells, immune cells, glomerular endothelium, principal and intercalated cells [79,80,81]. Transcriptome comparison revealed that current iPSC-derived organoids resemble first or second trimester fetal kidney tissue. Interestingly, early and late clusters of developing podocytes and proximal tubule cells were detected, indicating heterogeneous development [74,79,80,81,82,83,84]. On the protein level, physiological arrangement of the various nephron segments was shown using stainings for various podocyte markers (e.g., nephrin, podocalyxin, WT1), *Lotus Tetragonolobus* lectin to mark proximal tubules, and E-Cadherin minus and plus GATA3 to identify respectively distal tubules and developing collecting ducts [67,74,76,78]. Moreover, several proteins required for glomerular and tubular function were present. Organoid podocytes express a range of proteins required for glomerular function (e.g., nephrin, podocin, podocalyxin, synaptopodin) which are nearly absent in conventional 2D podocyte cell lines. Confirmed tubular transport proteins include megalin, cubulin, Na-K-Cl cotransporter 2, and calbindin-1 [67,74,76,79,80,81,82,83,84]. Stromal populations were identified as well. The expression of FOXD1 and MEIS1 indicated the presence of cortical and medullary interstitial cells and probably pericytes in close proximity to the endothelium [74,76,85].

The functionality of PSC organoids is less thoroughly investigated [86]. So far, proximal tubule endocytic receptor function was shown by dextran uptake [74]. Furthermore, the uptake of fluorescent methotrexate is suggestive of organic anion transporter function, although the expression of drug transport proteins was not detected [4,67,86]. Other proximal tubule functions and transport of electrolytes or water reabsorption in the more distal parts of the nephron were not yet shown.

Various novel strategies emerged to further characterize and mature organoids. High-throughput screens were developed that expedite improvement of differentiation in terms of growth factor concentrations, timing and duration. Minor concentration changes in factors such as CHIR99021 or FGF9 have major effects on the proportion of UB, MM, and early proximal and distal nephron cells [80,87]. To better understand and characterize complex cell fate dynamics of human kidney development in organoids, genetic tools were established during the past years [82,88,89,90,91,92]. Using a SIX2+ reporter line, it was shown that SIX2+ progenitor cells contribute to proximal nephron formation, but are not involved in collecting duct development [92]. In addition, SIX2:CITED1, MAFB:GATA3, and LRP2:HNF4α dual reporter lines were successfully generated to monitor podocyte, proximal tubule and collecting duct development [91]. Another approach is organoid implementation in microfluidics systems. Superfusion enhanced the number of endothelial vessels and improved podocyte characteristics [87,93]. Besides in vitro approaches, xenograft transplantation to mice resulted in improved maturation of organoid tissue (e.g., expression of Na-Cl cotransporter and aquaporin 2) [94,95]. Knowledge obtained from these studies is highly valuable to understand what signaling pathways are required to improve in vitro maturation. Taken together, recent developments in single cell RNA-sequencing combined with high-throughput (microfluidic) platforms, lineage tracing and transplantation with maturation in vivo are excellent combinations to acquire insights that advance organoid differentiation and reproducibility and permit detailed validation of new protocols.

#### 3.1.3. Applications

Organoids derived from iPSC allow detailed studies of the (patho)physiology of renal development, screening for compound nephrotoxicity or teratogenesis, and potentially implementation in renal replacement therapies. As extensively reviewed by Koning et al., various congenital disorders have been successfully studied using organoids, including polycystic kidney disease (PKD1, PKD2), congenital nephrotic syndrome (NPHS1), podocalyxin mutations, and nephronophthisis-related ciliopathy (IFT140) [96]. Other examples of disease modeling include the metabolic disease cystinosis and Mucin-1 kidney disease. Cystinotic organoids were established from patient-derived PSC and recapitulated typical pathophysiologic features, including elevated cystine levels and perturbed autophagy. Upon drug testing, the mTOR inhibitor everolimus was found to provide additional beneficial effects when combined with the current standard therapy cysteamine [97]. Another group developed kidney organoids from patients suffering from tubulo-interstitial disease caused by a mutation in the *MUC-1* gene. Mutant organoids showed Mucin-1 protein retention in vesicles of the early secretory compartment in kidney epithelial cells, which could be reversed by a small molecule that enabled the lysosomal degradation of the mutant protein. The molecular mechanism as well as the therapeutic effect of this compound were confirmed in organoids, patient cells and mice [98]. Renal fibrosis has been investigated as well. Lemos and co-workers resolved that interleukin-1β can induce a MYC-dependent metabolic switch that results in renal tubulointerstitial fibrosis in vivo and in vitro. In kidney organoids, interleukin-1β caused proximal tubule damage (upregulation of kidney injury molecule 1) and stimulated MYC-dependent activation of stromal cells and differentiation towards pro-fibrotic myofibroblasts [85]. A recent translational study focused on glomerulopathies. The authors found that the single cell transcriptome of glomerular cells (podocytes and parietal epithelial cells) in kidney organoids shares signatures with the developmental kidney. Interestingly, a similar signature (increased expression of LYPD1, PRSS23 and CHD6) was observed in glomerular tissue from kidney disease patients and observed in focal segmental glomerulosclerosis (FSGS) rats, suggesting reactivation of this developmental program upon injury [99]. These studies highlight the potential of kidney organoids to study the molecular mechanisms of renal disease, especially when combined with in vivo models and human kidney tissue. Organoid-based disease models are promising to aid the development of future therapies.

Another application is screening for adverse effects. Various nephrotoxic compounds were found to accumulate in organoids and inflict injury [86]. For example, adriamycin treatment caused dose-dependent podocyte damage and exposure to cisplatin and gentamycin induced kidney injury molecule-1 and apoptosis in the proximal segment [74,78,80,100]. These studies imply that organoids are potentially a valuable source for nephrotoxicity screening.

Kidney organoids derived from iPSC also hold promise as a source of renal tissue for regenerative medicine. Preliminary studies found that upon transplantation into mice, iPSC-derived kidney organoids engrafted, were vascularized by the host and became more mature [75,94,95]. Whether this approach may ultimately result in the development of a safe and full-scale kidney that can replace renal function remains to be determined. Another strategy is whole organ bioengineering, which has gained considerable interest during the past years. Cadaveric rat and human kidneys were decellularized and used as bioscaffolds. Recellularization of a rat kidney with human umbilical vein endothelial cells through the renal artery and rat neonatal kidney cells using the ureter resulted in the engraftment of both cell types and rudimentary renal function in vitro and in vivo [101]. Recently, a human bioscaffold was re-endothelialized with iPSC-derived endothelial cells via arteriovenous delivery. Endothelial cells were present in the cortex with expression levels comparable to normal human kidney. Moreover, hardly any occlusions where detected and the recellularized kidney was successfully perfused with blood [102]. Obviously, re-epithelialization with iPSC-derived renal epithelial cells would be the next step. However, this is challenging because it requires site-specific epithelial recellularization. Another strategy is generation of human kidneys by injection of human iPSC in blastocysts or the nephrogenic niche of kidney deficient large animals, though this approach is still in its infancy [5,103,104,105]. Altogether, this exciting work indicates that iPSC-derived kidney organoids may provide a novel source of donor organs in the future.

#### 3.1.4. Challenges

Despite the promising applications, iPSC organoids face several challenges. A major hurdle is the risk for tumor and teratoma formation, which interferes with experiments and use in regenerative medicine. Using retroviral transfection to generate iPSC poses a risk for tumorigenesis via interference with proto-oncogenes or tumor suppressor genes. Therefore, nowadays novel more subtle reprogramming methods (see Section 3.1.1) are used that reduce this risk, although these can have other drawbacks [106]. Furthermore, regardless of the method, reprogrammed cells frequently display genomic instability. This might be inherited from the cell of origin or caused by reprogramming or in vitro culture [107,108]. Another challenge is the presence of off-target cell types after differentiation. Single cell RNA-sequencing revealed that PSC-derived kidney organoids contain several non-renal cell types, including cardiac, reproductive, neuronal, undifferentiated and unidentified cells [79,80,109]. Undifferentiated and off-target cells pose a risk for teratoma formation, as became evident in a study where subcutaneous transplantation of PSC-derived nephron precursors to mice caused cartilage formation in some cases [95]. Strategies to reduce unwanted cell types are emerging. For example, tropomyosin receptor kinase B inhibition during differentiation resulted in a major reduction of neuronal cells and improved the amount of proximal tubule cells and podocytes [79].

A second issue that can interfere with experiments is variation between batches of organoids from the same iPSC line. Phipson and co-workers tested the reproducibility of their differentiation protocol (Little group) using single-cell RNA sequencing and reported that batch-to-batch variation significantly influenced nephron maturation and patterning, as well as the proportion of on- and off-target cell types. Interestingly, transcriptional congruence was shown between organoids from different iPSC lines within the same batch. This suggests that technical issues are the major source of variation and that comparisons between different organoids require a simultaneous differentiation work-up [110]. However, another group found that whilst cell types were reproducible across protocols, replicates and time points, cell proportions differed between four iPSC lines. This effect was mainly caused by off-target cells [109].

Maturation of PSC organoids also remains a challenge. Currently, organoids resemble first or second trimester fetal kidney [74,79,80,81,82,83,84]. This is related to the fact that culture of iPSC organoids is currently limited to a maximum of around 7 + 25 days, using air-liquid or suspension cultures [74,82,90,100]. Organoids beyond day 7 + 25 display divergent patterns of dysplasia such as cyst formation and mesenchyme expansion, which could be caused by off-target cells or a lack of nutrient and waste exchange. Another missing link could be the absence of immune cells, serum growth factors and endocrine signals [100]. Interestingly, organoids transplanted at the age of 7 + 18 days, successfully grew for additional 28 days in mouse kidneys. Transplanted organoids showed improved maturation of podocytes and tubular cells and a reduced number of off-target cells [94,109]. Further in vitro organoid maturation will be crucial to enhance the expression and function of proteins that are essential for adequate filtration, secretion and reabsorption along the nephron. Identifying the factors responsible for the improved differentiation in vivo will help to optimize in vitro protocols.

Despite the quite physiological organization at nephron level, generating a higher level of organization with regard to a common urinary collecting system and vasculature remains challenging [5]. Nonetheless, progress is made towards achieving these goals. For example, rodent organoids that share a common urinary collecting system were formed by co-culture of separately induced UB and MM together with stromal cells [76]. Elucidation of specific signals that contribute to branching morphogenesis and introduction of the required stromal populations could help to induce further UB branching and higher-order organization in human kidney organoids as well. In addition, a degree of glomerular vascularization was induced by the addition of VEGFA to the static cultures in vitro [80]. Moreover, when organoids (differentiated to pretubular aggregates) were exposed to perfusion shear stress in a microfluidic chip, vasculature abundance increased and glomerular wrapping and invasion of perfusable vessels was described. A mature glomerular basement membrane and functional filtration were not yet shown [93]. Other studies investigated organoid behavior in vivo and report that transplantation to mice resulted in vascularization with connection to the recipient vasculature [84,94]. Microfluidic platforms and lessons from xenografts hold promise to advance organoid vasculature and maturation.

### 3.2. Adult Stem or Progenitor Cell-Derived Kidney Tubuloids

#### 3.2.1. Sources of Adult Stem or Progenitor Cells

Organoid cultures from ASC were first established from the mouse small intestine in 2009. In vitro recreation of the intestinal stem cell niche using specific growth factors and a basement membrane gel that facilitates 3D growth allowed long-term expansion of LGR5+ intestinal stem cells [111]. Since then, ASC-derived organoids were established in rapid succession from many human organs as well, including the small intestine and colon, stomach, liver, pancreas, lungs, prostate, fallopian tube, bladder and recently the kidney [2,3,9]. A hallmark feature is that this approach allows long-term expansion of primary epithelium. In a similar fashion, ASC-derived tubuloids grow from primary renal epithelial cells collected from either kidney tissue (biopsies or nephrectomies) or in a rare and highly favorable non-invasive way from urine.

#### 3.2.2. Generation and Characterization of Adult Stem or Progenitor Cell-Derived Kidney Tubuloids

Tubuloids are 3D multicellular cultures that consist of adult kidney tubular epithelium. To generate tubuloids, primary renal epithelial cells are encapsulated in a basement membrane gel that allows 3D growth and cultured in a growth factor-rich medium. A stem cell/progenitor state is induced in these cells by stimuli such as Wnt amplification by R-spondin and activation of tyrosine kinase signaling by EGF and FGF. This results in proliferation and formation of heterogeneous 3D structures. Tubuloids during expansion express highly increased levels of CD24, CD44, CD133, SOX9 and Vimentin compared to primary kidney tissue, as well as decreased levels of transporters that mark differentiated cells, such as SLC34A1. Their proliferative capacity and expression profile that fits stemness and dedifferentiation indicate that tubuloids recapitulate the renal plasticity-based regeneration response in vitro. This allows exponential expansion of primary kidney epithelium for many months in a physiological way, without requiring genetic reprogramming or immortalization. Furthermore, tubuloids remain genetically stable in this period as shown by karyotyping and whole genome sequencing [9].

Similar to other ASC-derived organoids, tubuloids consist entirely of epithelium. Bulk and single cell RNA-sequencing as well as immunocytochemistry confirm that tubuloids contain a pure population of polarized proximal tubule, loop of Henle, distal tubule, and collecting duct epithelium. Podocytes and parietal epithelial cells were not detected. Transcriptomics and stainings also showed the presence of various important transporter proteins, including multidrug resistance-associated proteins 3 and 4, organic cation transporter 3, Na-K-Cl cotransporter 2, anion exchanger 1, and aquaporin 3. Furthermore, redifferentiation of tubuloids by withdrawal of growth factors that amplify Wnt signaling (R-spondin) and activate tyrosine kinase receptors (EGF, FGF) induced expression of the proteins calbindin-1 and uromodulin that are characteristic of differentiated distal tubule and loop of Henle cells [9].

The first proof of principle experiments show that tubuloid epithelium contains functional transporters. Tubuloids in 3D culture displayed P-glycoprotein-mediated efflux. Furthermore, tubuloids were integrated in the Organoplate^®^, an advanced microfluidic system for more in-depth characterization. The resulting tubuloid-on-a-chip proved leak-tight and capable of transepithelial transport by the concerted action of basolateral and apical transporters [9].

#### 3.2.3. Applications

Tubuloids hold great potential to model kidney disease and screen for effective drugs, because they can be rapidly established and accurately recapitulate the donor genotype and phenotype. So far, tubuloids were used to model infectious, hereditary, metabolic and malignant diseases. Infectious models were developed by infecting tubuloids with BK-virus (BKV), a pathogen that is mostly encountered in the bladder and kidneys of immunocompromised patients. Upon BKV infection, tubuloids displayed characteristic enlarged nuclei positive for viral antigens and actual viral particles were visualized in the nucleus. Viral replication was confirmed by qPCR and could be reduced using the drug cidofovir. Tubuloids were also successfully established from the urine of a patient with the genetic disease cystic fibrosis (CF). In contrast to healthy controls, CF tubuloids showed the characteristic lack of swelling after cystic fibrosis transmembrane conductance regulator (CFTR) activation by forskolin. Interestingly, when treated with the CFTR potentiator ivacaftor that also clinically benefitted this patient, tubuloid swelling was restored [9]. Our lab also found that tubuloids derived from urine of patients with the hereditary lysosomal storage disease cystinosis recapitulate the metabolic abnormalities characteristic of the disease, most notably cystine accumulation. These tubuloids can be used to test the effects of the current treatment cysteamine as well as novel treatments [112]. Tubuloids were also established from kidney tumor tissue to study malignant disease [9,113]. Moreover, tubuloid culture protocols were used to generate a large biobank containing tumor organoids from pediatric patients with various different kidney tumors. These tumor organoids closely mimic the histologic phenotype, genome, transcriptome and epigenome of the in vivo tumors. This makes these tumor organoids highly amenable for investigation of the pathophysiology and screening for sensitivity to chemotherapeutics to facilitate drug development and personalized medicine [114]. Drug efficacy screening was previously shown to correlate well with clinical outcomes in intestinal tumor organoids [115]. These divergent disease models and proof of principle drug efficacy screenings pave the way to personalized medicine in nephrology.

Tubuloids also proved useful for screening for nephrotoxicity, a common serious adverse effect of many therapeutic drugs. Exposure of the tubuloid-on-a-chip to the nephrotoxic drug cisplatin resulted in DNA damage and increased lactate dehydrogenase activity [9] (and unpublished data). Furthermore, the protocol used for chemotherapeutic efficacy screening in tumor organoids was modified to investigate nephrotoxicity in tubuloids in 3D culture [112,116]. Tubuloids can therefore be a promising tool for early selection of non-toxic compounds during drug development.

When seeded in the Organoplate^®^ organ-on-a-chip system, the perfusable tubular architecture allowed tubuloid epithelium to grow into a more physiological leak-tight and functional tubular structure [9]. This advanced system opens new opportunities, since it also permits the application of flow, implementation of various natural (e.g., collagen) matrices, and interaction with vasculature, stromal cell types, and epithelial cells from other organs. This enables for example exploration of the interplay between senescent epithelial cells, pericytes and endothelium in the pathogenesis of CKD. The combination of tubuloids and organ-on-a-chip technology allows studies of multi-tissue/organ interactions and high-throughput screening of renal epithelial function, nephrotoxicity and drug efficacy in a personalized fashion.

Finally, tubuloids are promising for regenerative nephrology. Transplantation and safety studies have not yet been performed. Nonetheless, tubuloids are an easily established source of expectedly not immunogenic nor oncogenic leak-tight and functional kidney epithelium, a very favorable profile for cell therapy and tissue-based replacement of kidney function. The fact that human and mouse ASC-derived colon organoids were successfully engrafted in damaged colons of mice recipients further fuels the promise of ASC-derived tubuloids in regenerative medicine [117,118].

#### 3.2.4. Challenges

To expand the exciting possibilities of tubuloids, several challenges need to be addressed. First of all, huge numbers of tubuloids are required for screenings of large libraries of thousands of compounds and for regenerative nephrology. Therefore, although tubuloid culture already permits long-term exponential growth of renal epithelium, further enhancement of expansion capacity is feasible. For this purpose, techniques such as the recently reported spinner flask method that robustly boosts the expansion of ASC hepatic organoids are an interesting starting point [119].

Secondly, podocytes and parietal epithelial cells were so far not detected in tubuloids [9]. Optimization of retrieval of these cell types (e.g., by refining tissue digestion or sorting these populations) and culture conditions could help to introduce these cell types in tubuloid cultures. Insights in podocyte regeneration in vivo can aid development of such protocols. However, although various studies present evidence for podocyte regeneration in mice, other studies describe a very limited regenerative capacity [120,121,122,123]. The latter could be an alternative explanation for the absence of these cells.

Although tubuloids express several markers of differentiation up to levels comparable to adult kidney tissue, some essential transporters are lowly expressed. This is not surprising since tubuloid culture conditions induce a proliferative and dedifferentiated stem cell state in renal epithelial cells. Redifferentiation by growth factor withdrawal was found to enhance expression of loop of Henle and distal tubule transporters and proteins [9]. These differentiation protocols require further improvement to grant control over tubuloid patterning and induce the expression of important transporters and enzymes required for disease modeling and regenerative medicine.

Application in regenerative nephrology requires organization of cell types similar to the nephron in vivo. Whereas PSC kidney organoids self-organize in near-physiological nephron-like structures, ASC tubuloids organize into cystic and dense structures that predominantly consist of one nephron segment each [9]. To guide the shape and orchestrate arrangement into more physiological nephron structures, tubuloids require environmental cues such as growth and differentiation factors, as well as physical guidance by biological or synthetical matrices. Furthermore, because tubuloids only consist of pure renal epithelium, introduction of glomerular structures, tubular vascularization and stroma requires co-culture with endothelial and stromal cells.

For clinical use in regenerative nephrology, the development of reproducible clinical grade synthetic matrices that support tubuloid-derived self-sustainable living biomembranes is also paramount. Currently, tubuloids are cultured in basement membrane gels derived from Engelbreth-Holm-Swarm sarcoma tissue from mice. These gels need to be replaced by biocompatible matrices that allow controlled tubuloid growth (epithelialization, maintenance, repair) with equal or superior efficiency and that provide connections to the recipient vasculature and/or urine drainage system.

## 4. Conclusions and Future Outlook

**Figure 4:**
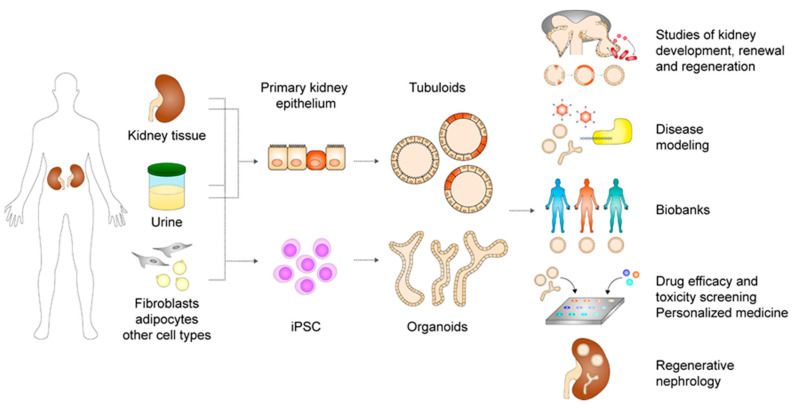
Establishment and applications of adult stem or progenitor cell-derived kidney tubuloids and induced pluripotent stem cell-derived kidney organoids. iPSC = induced pluripotent stem cell.

Kidney organoids and tubuloids are advanced in vitro models that open a window to a wide array of new possibilities for fundamental research and medicine (Figure 4). Whereas PSC organoids mimic nephrogenesis to give rise to highly complex in vivo-resembling kidney structures, ASC tubuloids model homeostasis and regeneration in the adult kidney and offer an extensively expandable autologous and genetically stable source of more mature epithelium. These are different and complementary approaches, each with their own specific applications.

First of all, both organoid types can elevate our understanding of kidney development, renewal and repair since they mimic these in vivo processes [124]. Organoids from PSC now permit in vitro modeling of human nephrogenesis and associated congenital abnormalities. The resulting insights can help to comprehend and treat congenital disorders. Tubuloids are in particular useful to unravel markers and molecular mechanisms of tubular turnover and repair, which can for example provide therapeutic targets that can ameliorate regeneration upon AKI and prevent progression to CKD. Techniques such as clustered regularly interspaced short palindromic repeats (CRISPR)-Cas9-induced gene knock-out and fluorescent reporter lines (e.g., those reported by Vanslambrouck et al.) combined with single cell RNA-sequencing will greatly facilitate such studies [91].

Secondly, organoids and tubuloids are widely used to model hereditary, infectious, metabolic, toxic and malignant diseases affecting the glomerulus or tubular nephron. The results are exciting and warrant extension to many more renal diseases. Because PSC organoids mimic nephrogenesis, they are especially well suited for modeling of teratogenic and developmental diseases. Tubuloids on the other hand are best qualified for diseases that manifest in the fully developed kidney and disorders of cell cycle regulation (e.g., senescence, cancer). Whereas tubuloids at this moment lack interstitium and a glomerular structure, PSC-based organoids contain stromal cells and relatively mature podocytes and allow modeling of stromal and glomerular diseases. On the other hand, tubuloids consist of polarized, leak-tight and more functional tubular epithelium and are therefore currently more suitable for studies of tubular transport and related diseases. Further maturation will better qualify PSC organoids for these purposes as well. Certain diseases involve more components than kidney cells and require co-cultures, for example addition of immune cells to model auto-immune glomerulonephritis and co-culture with vasculature and pericytes to model transition to CKD.

Furthermore, organoid and tubuloid-based disease models allow in vitro screening of wide arrays of (pharmacological) compounds to predict therapeutic efficacy and nephrotoxicity. At this moment, novel treatments for various diseases are tested in organoid and tubuloid disease models and show promising results [97,98,112]. Various nephrotoxicity studies were performed as well in both organoid types. Organoids and tubuloids much closer reflect the in vivo nephron than traditional cell lines and allow studies of human tissue in a high-throughput fashion in contrast to animal models. Efficacy and toxicity screenings in these advanced in vitro models are therefore expected to significantly contribute to preclinical drug development in the near future. Therapeutic interventions other than medication can be tested as well. Exciting state-of-the-art CRISPR-based techniques such as double stranded break-free base editing and prime editing warrant a new surge of studies investigating gene therapy for genetic disease. Organoid- and tubuloid-based disease models are an excellent system to test such interventions [125,126].

Promising translational developments also include the upscaling of disease models to biobanks and personalized medicine. Biobanks of tubuloids as described by Calandrini et al. allow drug screening and validation in a large group of patients with a particular disease to discover interventions that benefit patients with that disease in general [114]. Moreover, organoids and tubuloids can be used for personalized medicine. This is not a distant prospect, since ASC-based rectal organoids are currently already used in Dutch hospitals to predict therapeutic response in CF patients [127]. Because tubuloids can be easily and rapidly established from autologous cells with high efficiency, they offer the option to generate large biobanks and facilitate personalized medicine with relative ease. To realize clinical translation, the major next challenge is to validate in vitro drug screenings by correlation to actual clinical outcomes.

Finally, both organoids and tubuloids are expected to play a major role in the quest for cell therapy and tissue-based renal replacement strategies to treat kidney failure. Organoids from PSC form near-physiological nephron structures and vascularize and mature to some extent in preliminary xenograft studies. Tubuloids are less organized and currently lack a glomerulus, but are easily established, more functional, and have a highly favorable safety profile.

In conclusion, kidney organoids and tubuloids form a long sought-after source of human patient-specific renal tissue that opens a new chapter in kidney research and clinical nephrology. Their anticipated role in both the prevention and treatment of kidney diseases is an exciting prospect.
